# Coenzyme Q-10 improves preservation of mitochondrial functionality and actin structure of cryopreserved stallion sperm

**DOI:** 10.1590/1984-3143-AR2020-0218

**Published:** 2021-03-31

**Authors:** Renata Lançoni, Eneiva Carla Carvalho Celeghini, Valdemar De Giuli, Carla Patricia Teodoro de Carvalho, Gabriela Bertaiolli Zoca, Laura Nataly Garcia-Oliveros, Leonardo Batissaco, Letícia Zoccolaro Oliveira, Rubens Paes de Arruda

**Affiliations:** 1 Departamento de Reprodução Animal, Universidade Federal de Uberlândia, Uberlândia, MG, Brasil; 2 Departamento de Reprodução Animal, Universidade de São Paulo, Pirassununga, SP, Brasil; 3 Central Internacional de Reprodução Equina Rancho das Américas, Porto Feliz, SP, Brasil; 4 Departamento de Clínica Veterinária e Cirurgia, Universidade Federal de Minas Gerais, Belo Horizonte, MG, Brasil

**Keywords:** antioxidant, cytoskeleton, cryopreservation, equine, sperm quality

## Abstract

Coenzyme Q-10 (CoQ-10) is a cofactor for mitochondrial electron transport chain and may be an alternative to improve sperm quality of cryopreserved equine semen. This work aimed to improve stallion semen quality after freezing by adding CoQ-10 to the cryopreservation protocol. Seven saddle stallions were utilized. Each animal was submitted to five semen collections and freezing procedures. For cryopreservation, each ejaculate was divided in three treatments: 1) Botucrio® diluent (control); 2) 50 μmol CoQ-10 added to Botucrio® diluent; 3) 1 mmol CoQ-10 added to Botucrio® diluent. Semen batches were analyzed for sperm motility characteristics (CASA), plasma and acrosomal membranes integrity and mitochondrial membrane potential (by fluorescence probes propidium iodide, Hoechst 33342, FITC-PSA and JC-1, respectively), alterations in cytoskeletal actin (phalloidin-FITC) and mitochondrial function (diaminobenzidine; DAB). The 1 mmol CoQ-10 treatment presented higher (P<0.05) amount (66.8%) of sperm cells with fully stained midpiece (indicating high mitochondrial activity) and higher (P<0.05) amount (81.6%) of cells without actin reorganization to the post-acrosomal region compared to control group (60.8% and 76.0%, respectively). It was concluded that the addition of 1 mmol CoQ-10 to the freezing diluent was more effective in preserving mitochondria functionality and cytoskeleton of sperm cells submitted to cryopreservation process.

## Introduction

Most stallions were genetically selected based on their pedigree and athletic performance rather than for fertility potential. As a result, semen quality and fertility of these animals are quite variable and some stallions may have poor quality sperm (Allen and Wilsher, 2011), which leads to great difficulty of large-scale use of equine cryopreserved semen (Al-Essawe et al., 2018). Hence, many studies have been performed to improve stallion semen quality in order to increase the efficiency of reproductive biotechniques (Affonso et al., 2017; Lançoni et al., 2018; Nascimento et al., 2008; Tsunoda et al., 2015).

Peña et al. (2015) demonstrated that mitochondria are key structures in sperm function and may undergo major changes during cryopreservation. Important differences are observed between species on intracellular energy generation. Spermatozoa of several mammalian species (humans, rats and bulls) mainly produce ATP by glycolysis, unlike what occurs in stallions which evidences indicate that mitochondrial oxidative phosphorylation, that is more efficient in ATP generation as compared with glycolysis, is the main source of ATP production in equine sperm (Ortega-Ferrusola et al., 2010; Gibb et al., 2014). Hence, mitochondrial functionality is especially important for stallion sperm.

Coenzyme Q-10 (CoQ-10) is a lipid molecule present in mammalian cells, which is capable of promoting energy generation through the exchange of electrons and protons that occurs during electron transport chain in the internal mitochondrial membrane (Lewin and Lavon, 1997). Due to its direct action in intracellular energy production, CoQ-10 is involved with several phisiological processes (Littarru and Tiano, 2010). In addition to its role in cellular bioenergetics, it is considered to be the only liposoluble antioxidant endogenously synthesized (Turunen et al., 2004).

CoQ-10 acts on the bioenergetic function of the cell and also has an antioxidant effect that may indicate possible involvement in male infertility because these factors are directly related to sperm viability, and a spermatozoon without energy or in oxidative stress, probably will not be successful in fertilization. It is known that large number of mitochondria are required due to the high sperm energetic demand for motility (Fawcett, 1975). On the other hand, protection of mitochondrial membranes against oxidative stress is fundamental for maintenance of sperm integrity (Balercia et al., 2009). One of the mitochondria assessment methods, a cytochemical test was developed by Hrudka (1987). The enzyme cytochrome C-oxidase (CCO) plays a key role in the process of sperm metabolism being closely related to cellular respiration and mitochondrial energy generation.

Actin, an element of the cytoskeleton, plays a crucial role in the regulation of cellular shape, migration and interaction with extracellular matrices (Correa et al., 2007; Liu et al., 2005). The presence of actin in sperm tail is important for sperm motility regulation (Dai et al., 2015) and actin polymerization is important for the onset of sperm motility during post-testicular maturation (Lin et al., 2002).

The cellular cytoskeleton can be damaged when submitted to procedures involving changes in osmolarity and cell volume, as occurs in cryopreservation (Correa et al., 2007). The actin network undergoes rearrangements in response to osmotic stress and it has been proposed that plasma membrane defects are caused by loss of actin structure (Correa et al., 2007).

During the formation of F-actin polymers (a process also known as polymerization), actin binds to an ATP molecule and is hydrolyzed to ADP (Holmes et al., 1990). Interaction of ATP with ATPases located in the dynein arms of microtubule pairs promotes their slippage and flagellar movement (Eddy et al., 2003). Hence, it is possible to note the importance of cytoskeleton function in sperm cells and its relation with mitochondrial activity and ATP production.

Therefore, it was hypothesized that the addition of CoQ-10 to the diluent used for semen cryopreservation may improve post-thawing sperm characteristics of stallions. The objective of this work was to assess sperm motility, plasma and acrosomal membrane integrity and mitochondrial membrane potential of cryopreserved equine sperm, focusing on the performance of CoQ-10 in sperm mitochondrial functionality and cytoskeleton structure.

## Material and methods

### Management of animals, semen collection and prefreezing laboratory procedures

Seven adult saddle stallions were used in the present study. Five ejaculates from each were collected (n=35). Penis was cleaned before each collection, and semen was collected with artificial vagina (Botucatu model; Botupharma, Botucatu, SP, Brazil). Procedures described in this study were in agreement with the Bioethics Committee of the FMVZ/USP, protocol number 9044141216.

Immediately after collection, semen was filtered to separate the sperm rich part from the gel. Sperm concentration analysis was performed using Nucleo Counter®, (ChemoMetec, Denmark). The cassette was loaded by pressing the white piston down to the top of the cassette, which creates a partial vacuum in the flow system. The tip of the cassette was immersed into the sample and the piston was pressed, resulting in sample being loaded into the flow system. Approximately 60µl was loaded into the flow system filling the first part of the channel in the cassette. The liquid is transferred to the measurement chamber when the cassette is inserted in the NucleoCounter®. Then, semen was diluted using equal volume (1:1) of a commercial skim milk-based extender (Max Sêmen®), aliquoted into 15 mL tubes and centrifuged at 500 x *g* for 12 minutes. After centrifugation, the supernatant was removed and sperm concentration was adjusted to 200x10^6^ sperm/mL in the respective diluents (control or treated), as detailed below.

### Extender treatments

**Figure 1:**
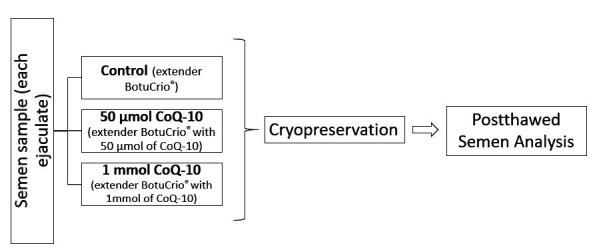
Representative scheme of treatments used.

Each ejaculates were divided into three treatments, being control (extender BotuCrio® - Botupharma, Botucatu, SP, Brazil) and the same extender with two different concentrations of CoQ-10 (Sigma-Aldrich® - C9538) that were added (50 µmol of CoQ-10 and 1 mmol of CoQ-10) (Figure 1). These two chosen concentrations of CoQ-10 were based on literature review, especially in papers (Lewin and Lavon, 1997; Rossi et al., 2016). Hence, the treatments were denominated as control (CONT), 50 µmol CoQ-10 and 1 mmol CoQ-10.

### Cryopreservation process

As previously described, semen was centrifuged and suspended in the specific extender (control or treatments) at a concentration of 200x10^6^ sperm/mL. Then, samples were packed into 0.50 mL semen straws, carefully sealed and identified for each treatment.

Semen cryopreservation was performed using TK 3000® equipment (TK Freezing Technology LTDA, Uberaba, MG, Brazil). Straws were refrigerated at 5 °C, using the cooling rate of -0.5 °C/min. Hence, the samples remained for 30 minutes at 5 °C in stabilization (equilibrium time). Then, the freezing curve was performed at a rate of -20 °C/min from 5 to -120 °C (Lançoni et al., 2018). Then straws were immersed in liquid nitrogen (-196 °C).

### Postthawed semen analysis

For assessment of frozen-thawed semen, two straws were thawed for each semen batch from each treatment (CONT, 50 µmol CoQ-10 and 1 mmol CoQ-10). Thawing procedure was carried out in a water bath at 37 °C for 30 seconds.

#### Sperm motility

Sperm motility was evaluated using CASA system (*Computer Assisted Sperm Analisys;* Hamilton Thorne Research Motility Analyser, HTM-IVOS, versão 12.3, Hamilton Thorn Research, Beverly, Massachusetts, USA). The setup was previously adjusted for analysis of equine spermatozoa. Before evaluation, sperm samples were diluted at a final concentration of 40x10^6^sperm/mL with the respective diluents (CONT, 50 µmol CoQ-10 and 1 mmol CoQ-10). Next, 10 µL of diluted semen was deposited on the Makler® chamber (Self-Medical Instruments, Haifa, Israel) and the following characteristics were analyzed: total motility (TM, %), progressive motility (PM, %) and rapid cells (RAP, %).

#### Assessment of plasma, acrosomal, and mitochondrial membranes

Initially, 150 mL of semen was diluted in the TALP *sperm* medium at a concentration of 25x10^6^ sperm/mL in a microcentrifuge tube. Then, 3 µL of propidium iodide (PI 0.5 mg/mL), 2 µL of Hoechst 33342 (0.5 mg/mL), 6 µL of 5,5’,6,6’-tetrachloro-1,1’,3,3’ tetraethylbenzimidazolyl-carbocyanine iodide (JC-1, 153 µmol), and 80 µL of Pisum sativum agglutinin conjugated to fluorescein isothiocyanate (FITC-PSA 100 µg/mL) were added to the sample. The sample was then incubated for 8 minutes at 37 °C in the dark.

Then, a 4 µL aliquot was evaluated using 1000X magnification under epifluorescence microscopy (Eclipse Model Ni-U 80i; Nikon, Tokyo, Japan) with triple filter (D/F/R, C58420) featuring the UV-2E/C (excitation 340e380 nm and emission 435e485 nm), B-2E/C (excitation 465e495 nm and emission 515e555 nm), and G-2E/C (excitation 540-525 nm and emission 605e655 nm).

A total of 200 cells were counted and classified according to their staining patterns in percentage of cells with intact plasma membrane (IPM); percentage of cells with intact acrosomal membrane (IA); percentage of cells with high mitochondrial membrane potential (HMMP), and percentage of fully intact cells (sperm cells presenting intact plasma and acrosomal membranes and HMMP [PIAIH]) (adapted from Celeghini et al., 2007).

#### Assessment of mitochondrial functionality

Mitochondrial activity assessed by DAB technique is based on cytochrome C oxidation of 3,3'-Diaminobenzidine (DAB), including CCO, where polymerized reagents are deposited at the reaction sites, i.e., the mitochondria (Hrudka, 1987). Hence, an aliquot (25 μL) of semen was incubated with 25 μL of DAB (Diaminobenzidine - 1 mg/mL in PBS) at 37 °C for one hour. After incubation, semen smears were fixed in 10% formaldehyde for 10 min.

The slides were air-dried in the dark and 200 cells from each sample were counted under 1000X magnification (optical microscopy) and classified into 4 classes as described by Hrudka (1987):

DAB 1: sperm cells with fully stained midpiece and fully active mitochondria, indicating high mitochondrial activity. DAB 2: sperm cells with mostly stained mitochondria (>50%), indicating medium to high mitochondrial activity. DAB 3: sperm cells with lower than half of stained mitochondria (<50%), indicating low mitochondrial activity. DAB 4: sperm cells with completely unstained midpiece, indicating absence of mitochondrial activity.

#### Assessment of actin filaments alterations - Cytoskeleton

To analyze actin cytoskeleton, phalloidin (FITC-conjugated) was used (Phalloidin-FITC; Sigma-Aldrich code P5282). The protocol for fluorescence staining of actin filaments was adapted from Brener et al. (2003) and Macías García et al. (2012). Protocol modifications were previously validated by our research group for this experiment.

Hence, a 25 µL alíquot of each sample was added to 150 μL of Tris buffered saline (TBS 10x, pH adjusted to 7.4). Smears from diluted semen were made on slides previously treated with poly-L-lysine and fixed in 5% formaldehyde for 10 min. Then, smears were washed in PBS and permeabilized using cooled acetone for 10 min and air-dried at room temperature. After permeabilization, cells were again washed in TBS containing 1% of BSA and further incubated for 10 min. Finally, cells were stained with 5 µL of phalloidin-FITC (2 mmol in DMSO), coverslipped (coverslip size 24 × 60 mm) and incubated for 60 min at room temperature (22 °C) in the dark. After incubation the cells were examined using 1000X magnification under epifluorescence microscopy (Eclipse Model Ni-U 80i; Nikon, Tokyo, Japan). Two hundred cells were counted for each sample and classified according to brightness intensity (high or low) in the post-acrosomal region, based on the patterns described by Brener et al. (2003) and Macías García et al. (2012).

### Statistical analysis

Statistical analyses were performed using SAS software (version 9.3; SAS Institute, Inc., Cary, NC). Previously, data were tested for normality (ShapiroeWilk test). The variables that did not comply with statistical premises were transformed. Comparisons among treatments were performed by MIXED procedure of SAS, using treatment as a fixed effect and stallions and ejaculate within each stallion as random effects. Differences between treatments were obtained using Tukey test. The probability of P ≤ 0.05 was considered as significant, and the probability between P ˃ 0.05 e ≤ 0.10 was considered as statistical tendency. Data are present as mean ± standard error of the mean (S.E.M.).

## Results

### Characteristics of sperm motility

In general, no significant effects were observed among treatments on postthawed sperm motility (Table 1).

**Table 1:** Mean ± S.E.M. values of motility characteristics (CASA) in cryopreserved stallion sperm submitted to different treatments with CoQ-10 added to freezing extender.

**Sperm Characteristics (%)**	**Treatments**			**Mean**
	**Control**	**50 µmol CoQ-10**	**1 mmol CoQ-10**	
**Total motility**	58.80 ± 3.68	61.48 ± 3.87	60.08 ± 3.83	60.12 ± 2.17
**Progressive motility**	40.02 ± 3.37	40.51 ± 3.45	40.11 ± 3.50	40.21 ± 1.97
**Rapid cells**	50.65 ± 3.93	52.77 ± 4.13	51.97 ± 4.16	51.80 ± 2.33

### Plasma and acrosome membranes integrity and mitochondrial membrane potential

The CoQ-10 treatment, regardless of concentration, increased (P˃0.05 and ≤ 0.10) the percentage of HMMP cells as well as the population of sperm with intact plasma membrane, intact acrosome and high mitochondrial membrane potential (PIAIH), as demonstrated in Table2.

**Table 2:** Mean ± S.E.M. values of sperm cells with intact plasma membrane (IPM); acrosomal integrity (IA); high mitochondrial membrane potential (HMMP); and sperm cells presenting intact plasma and acrosomal membranes and HMMP (PIAIH) in cryopreserved stallion semen submitted to different treatments of CoQ-10 added to freezing extenders.

**Sperm Characteristics (%)**	**Treatments**			**Mean**
	**Control**	**50 µmol CoQ-10**	**1 mmol CoQ-10**	
**IPM**	30.58 ± 1.84	32.18 ± 1.54	32.42 ± 1.52	31.73 ± 0.94
**IA**	75.15 ± 1.20	71.78 ± 1.72	74.15 ± 1.84	75.18 ± 0.80
**HMMP**	26.57 ± 1.93^b^	28.48 ± 1.84^a^	28.8 ± 1.84^a^	30.90 ± 0.90
**PIAIH**	29.00 ± 1.73^b^	31.24 ± 1.45^a^	31.47 ± 1.52^a^	30.57 ± 0.90

^a,b^ Different letters on the same row indicate statistical tendency (P ˃ 0.05 and ≤ 0.10) by the Tukey test.

### Mitochondrial functionality

**Figure 2:**
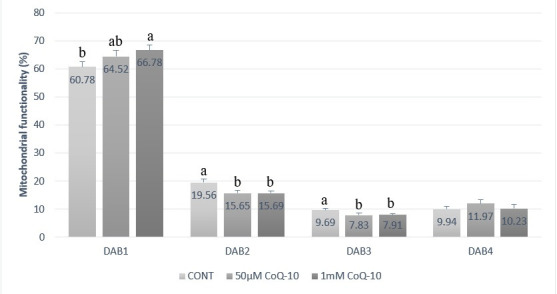
Means (± S.E.M.) of mitochondrial functionality from stallion cryopreserved sperm submitted to different CoQ-10 treatments. ^a,b^ Different letters on the bars indicate statistical difference (P < .05) by the Tukey test. DAB 1: sperm cells with fully stained midpiece, indicating high mitochondrial activity. DAB 2: sperm cells with mostly stained midpiece (>50%), indicating medium to high mitochondrial activity. DAB 3: sperm cells with lower than half of stained midpiece (<50%), indicating low mitochondrial activity. DAB 4: sperm cells with completely unstained midpiece, indicating absence of mitochondrial activity.

Regarding to mitochondrial functionality by DAB technique, it was observed that 1 mmol CoQ-10 treatment presented higher percentage of cells classified as DAB 1 (sperm with fully stained midpiece), indicating high mitochondrial activity (Figure 2). In addition, both CoQ-10 treatments (50 µmol and 1 mmol) demonstrated lower percentage of DAB 3 cells (sperm with low mitochondrial activity) compared to control group. No statistical differences were observed for DAB 4 classification (Figure 2).

### Reorganization of cytoskeletal actin filaments

**Figure 3:**
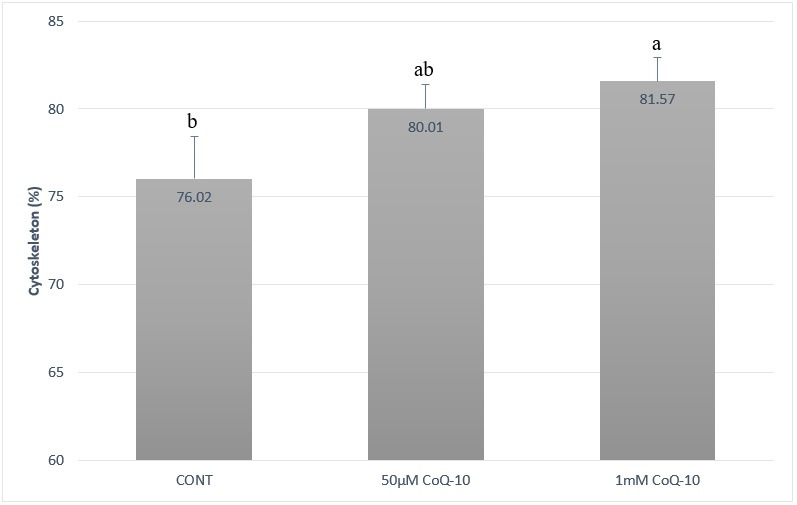
Mean (± S.E.M.) of sperm cells without actin reorganization to the post-acrosomal region of cryopreserved stallion semen submitted to different CoQ-10 treatments. ^a,b^ Different letters on the bars indicate statistical difference (P < .05) by the Tukey test.

Assessment of sperm with phalloidin-FITC fluorescence probe demonstrated that 1 mmol CoQ-10 treatment efficiently preserved cellular cytoskeleton during the cryopreservation process, exhibiting higher percentage of cells without actin reorganization to the post-acrosomal region after thawing. No diference was observed for 50 μmol CoQ-10 treatment compared to control group (Figure 3).

## Discussion

This study evaluated the effect of CoQ-10 on cellular bioenergetics, cytoskeleton, membranes integrity and sperm motility. The addition of CoQ-10 into freezing extender proved to be beneficial to cryopreserved equine semen, by preserving mitochondrial functionality and actin organization of sperm cells when used at the concentration of 1mmol.

CoQ-10 perform essential functions in the human body. It is responsible for electrons and protons transport to energy production, leading to ATP synthesis within mitochondrial membrane. Therefore, overall cellular energy production depends on CoQ-10 availability. Concerning the spermatozoa, CoQ-10 is mostly concentrated in the midpiece being used for ATP-dependent processes such as sperm motility (Lewin and Lavon, 1997). Moreover, ubiquinol (a reduced form of CoQ-10) acts as a strong antioxidant in several biological systems (such as membranes and lipoproteins) by protecting them from hydroperoxides formation and lipid peroxidation (Alleva et al., 1997; Bentinger et al., 2007). These important CoQ-10 properties suggests superior preservation (statistical tendency) of sperm membrane characteristics for the treated groups of present study, where higher mitochondrial membrane potential (HMMP) and fully intact cells (PIAIHM) were observed compared with control group (Table 2). No effect of CoQ-10 were observed for sperm motility parameters assessed by CASA (Table 1).

Results of this particular study demonstrated that using 1 mmol of CoQ-10 preserved mitochondrial activity of cryopreserved equine sperm when compared to the control group (Figure 2). This was probably due to the direct action of CoQ-10 on electron transport chain of mitochondrial membrane, improving and activating mitochondrial activity.

It has been stated that actin cytoskeleton may be damaged over the freezing process because cytoskeletal elements are sensitive to temperature variations, especially during refrigeration and cryopreservation (Watson, 1995, 2000). Sperm-cooling process affects actin organization increasing its intensity in the post-acrosomal region (Watson, 2000) and cellular refrigeration results in premature depolymerization of actin filaments in other cell types (Hall et al., 1993).

Actin filaments reorganization that occurs in sperm post-acrosomal region during cooling and freezing procedures (Watson, 1995, 2000; Flores et al., 2010) is related to extender osmolarity and temperature changes (Macías García et al., 2012). Therefore, it is implied that lower reorganization of actin filaments correspond to reduced cellular changes and sperm injuries. According to present results, it was possible to observe that control samples presented higher percentage (24.0%) of sperm cells with damaged cytoskeleton (i.e., cells presenting actin reorganization and displacement to the post-acrosomal region) whereas the 1 mmol CoQ-10 group presented lower amount (21.5%) of sperm cells with damaged actin cytoskeleton (Figure3). To the best of our knowledge, this study was the first demonstrating that sperm cytoskeleton was better preserved during cryopreservation of equine semen with the addition of CoQ-10 at 1 mmol concentration.

As previously described, actin cytoskeleton holds important role in the regulation of cell volume, sperm motility, plasma membrane integrity, acrosomal reaction and sperm capacitation. Thus, disclosing a tool that assists cytoskeleton preservation over cryopreservation process may be especially useful to progress and improvement of equine semen biotechnology.

## Conclusion

It was concluded that mitochondria functionality and actin cytoskeleton of sperm cells submitted to cryopreservation process is preserved most effectively by addition of CoQ-10 at 1 mmol concentration to freezing extender when compared to the control group, without CoQ-10.
